# Imaging of supratentorial intraventricular masses in children: a pictorial review—part 2

**DOI:** 10.1007/s00234-023-03253-3

**Published:** 2023-12-12

**Authors:** Fabricio Guimaraes Goncalves, Mario E. Mahecha-Carvajal, Aishwary Desa, Harun Yildiz, Jawabreh Kassem Talbeya, Luz Angela Moreno, Angela N. Viaene, Arastoo Vossough

**Affiliations:** 1 Radiology Department, Children´s of Alabama, Alabama, USA; 2 University of the Andes, Bogotá, Colombia; 3 Drexel University College of Medicine Philadelphia, Philadelphia, PA USA; 4 Department of Radiology, Dortcelik Children’s Hospital, Bursa, Turkey; 5 Bnai-Zion Medical Center, Haifa, Israel; 6 Pediatric Imaging, Department of Radiology, Fundación Hospital La Misericordia, Universidad Nacional de Colombia, Bogotá, Colombia; 7 Perelman School of Medicine, University of Pennsylvania, Philadelphia, PA USA; 8 Radiology Department, Children’s Hospital of Philadelphia, Philadelphia, PA USA; 9 Pathology Department, Children´s Hospital of Philadelphia, Philadelphia, USA

**Keywords:** Supratentorial intraventricular masses, Magnetic resonance imaging, Computed tomography, World Health Organization Classification of Tumors

## Abstract

**Purpose:**

This article is the second in a two-part series aimed at exploring the spectrum of supratentorial intraventricular masses in children. In particular, this part delves into masses originating from cells of the ventricular lining, those within the septum pellucidum, and brain parenchyma cells extending into the ventricles. The aim of this series is to offer a comprehensive understanding of these supratentorial intraventricular masses, encompassing their primary clinical findings and histological definitions.

**Methods:**

We conducted a review and analysis of relevant epidemiological data, the current genetics/molecular classifications as per the fifth edition of the World Health Organization (WHO) Classification of Tumors of the Central Nervous System (WHO CNS5), and imaging findings. Each supratentorial intraventricular mass was individually evaluated, with a detailed discussion on its clinical and histological features.

**Results:**

This article covers a range of supratentorial intraventricular masses observed in children. These include colloid cysts, subependymal giant cell astrocytomas, ependymomas, gangliogliomas, myxoid glioneuronal tumors, central neurocytomas, high-grade gliomas, pilocytic astrocytomas, cavernous malformations, and other embryonal tumors. Each mass type is characterized both clinically and histologically, offering an in-depth review of their individual imaging characteristics.

**Conclusion:**

The WHO CNS5 introduces notable changes, emphasizing the vital importance of molecular diagnostics in classifying pediatric central nervous system tumors. These foundational shifts have significant potential to impact management strategies and, as a result, the outcomes of intraventricular masses in children.

## Introduction

This is **part 2** of a two-part series on supratentorial intraventricular masses in children (Table 1). This part aims to deliver a comprehensive overview of both common and rare masses originating from cells that constitute the ventricular lining, cells within the septum pellucidum, and cells from the brain parenchyma that result in masses projecting into the ventricles. The spectrum of supratentorial intraventricular masses discussed includes colloid cysts, subependymal giant cell astrocytomas (SEGA), ependymomas, gangliogliomas, myxoid glioneuronal tumors, PDGFRA p.K385-mutant (MGNT), central neurocytomas (CN), high-grade gliomas (HGG), pilocytic astrocytomas (PA), cavernous malformations, and other embryonal tumors.

**Table 1:** Supratentorial intraventricular masses based on their cells of origin. Lesions that arise from cells within the CPs are discussed in Part 1, while lesions originating from the ventricular lining, septum pellucidum, and brain parenchyma discussed in Part 2

Choroid Plexus	Ventricular Lining	Septum Pellucidum	Brain Parenchyma
Primary Choroid Plexus Tumors	Colloid Cyst	Central Neurocytoma	High-grade Glioma / GBM
Choroid Plexus Hyperplasia	SEGA	High-grade Glioma / GBM	Pilocytic Astrocytoma
Choroid Plexus Cyst	Ependymoma	Pilocytic Astrocytoma	Ganglioglioma
Choroid Plexus Xanthogranuloma	Ganglioglioma	ATRT	ATRT
ATRT	Myxoid Glioneuronal Tumor	Cavernous Malformation	Cavernous Malformation
Meningioma	ATRT	Myxoid Glioneuronal Tumor	Other Embryonal Tumors
AVM	AVM	Other Embryonal Tumors	
Metastasis*		AVM	

*ATRT* Atypical teratoid rhabdoid tumor; *AVM* Arteriovenous malformation; *SEGA* Subependymal giant cell astrocytoma; *GBM* Glioblastoma

## Colloid cyst

### Background

Colloid cysts are rare intracranial lesions, affecting roughly three out of every million individuals annually. They are predominantly found in the rostral region of the third ventricle. While the clinical and imaging characteristics of colloid cysts are well established, their origins and the elements influencing their imaging features remain areas of continued discussion [1]. These cysts are predominantly identified in individuals in their third and fifth decades of life [2].

### Pathology

**Fig. 1:**
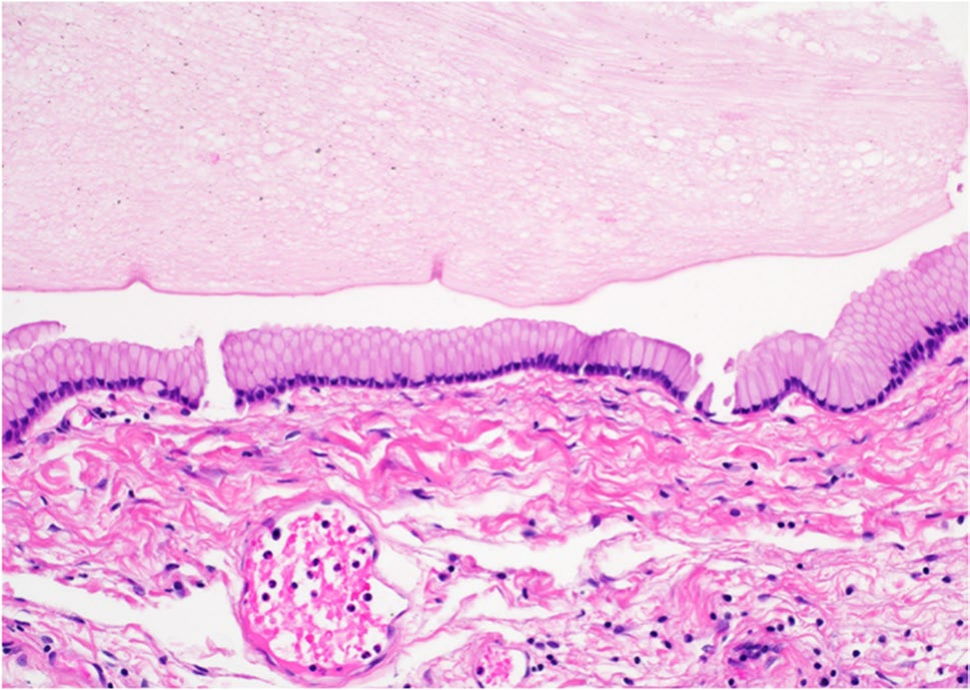
Colloid cyst: the image shows a cyst lined by columnar epithelium overlying fibrous stroma (bottom of the image) with abundant mucin contents (top of the image). H&E stain, 20 × magnification

Colloid cysts are unilocular round structures filled with a viscous material. The cell wall is unicellular and typically composed of columnar epithelium, which may or may not be ciliated. They exhibit the presence of hypocellular and fibrous stroma [3] (Fig. 1).

### Clinical presentation

Colloid cysts may present with intermittent, self-resolving, and nonspecific symptoms. These cysts can induce symptoms by obstructing the flow of cerebrospinal fluid (CSF), often resulting in intermittent episodes of headache, accompanied by vomiting, transient diplopia, and blurred vision. The episodic and paroxysmal nature of these symptoms can be indicative of colloid cysts. Symptoms may begin abruptly and be followed by periods of complete remission which can last from days to several weeks, or even months [1].

### Key diagnostic features

The primary imaging finding is a rounded mass located in the anterior third ventricle. The imaging appearance of colloid cysts can differ, mainly based on the cyst viscosity or cholesterol content. Furthermore, this cystic content plays a crucial role in determining the success of treatment [1].

### Computerized tomography

On computed tomography (CT), most colloid cysts appear hyperdense relative to the surrounding brain tissue, with hypo- or iso-dense cysts are less common. These cysts might also have a thin enhancing capsule. If they lead to hydrocephalus, CT may reveal periventricular hypodensity and varying degrees of lateral ventricle enlargement, resulting from elevated intraventricular pressure and periventricular white matter interstitial edema [1, 2, 4].

### Magnetic resonance imaging

**Fig. 2:**
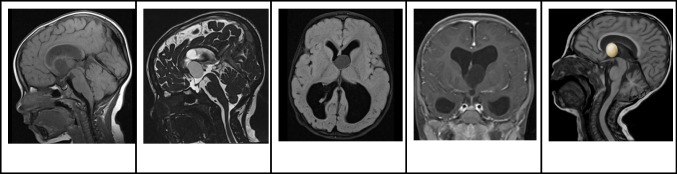
Colloid cyst in a 2-year-old boy with headaches and signs of increased intracranial pressure. **A** Sagittal T1-weighted image shows an oval-shaped well-defined hypointense nodule in the anterior third ventricle causing moderate hydrocephalus **B** Sagittal high resolution heavily T2-weighted image shows that the nodule has smooth margins and lower signal than the CSF in keeping with a cyst with proteinaceous material. **C** Axial FLAIR image shows that the anterior third ventricular cyst is not associated with edema or parenchymal invasion. **D** Coronal contrast-enhanced T1-weighted image shows that the cyst has no signs of enhancement. **E** Illustration representing the well-defined appearance of colloid cysts, which are typically located in the anterior third ventricle, commonly associated with obstructive hydrocephalus

The magnetic resonance imaging (MRI) characteristics of colloid cysts can differ widely based on the cyst content. Around two-thirds of colloid cysts appear hyperintense relative to brain tissue on T1-weighted imaging (WI), while the rest show either isointensity or hypointensity. On T2-weighted images, most colloid cysts are isointense to the brain, with some exhibiting peripheral rim enhancement. Notably, colloid cysts do not exhibit any diffusion restriction (Fig. 2) [1, 2, 4].

## Subependymal giant cell astrocytoma

### Background

Per the 2021 World Health Organization (WHO) Classification of Tumors of the CNS (WHO CNS5), subependymal giant cell astrocytomas (SEGA) are classified as grade 1, benign, non-infiltrative brain lesions [5]. Although commonly associated with tuberous sclerosis complex (TSC) [6], occurrences of SEGA without TSC are extremely rare [7].

TSC is an autosomal dominant neurocutaneous disorder, characterized by benign lesions in various organs, especially the brain, kidneys, liver, skin, heart, and lungs. Notably, TSC is marked by the presence of cortical tubers and subependymal nodules (SENs) within the brain. SEGAs, defined as circumscribed astrocytic gliomas, occur in about 10 to 20% of TSC individuals. These tumors often manifest during the second decade of life, predominantly arising near the foramen of Monro in the lateral ventricles. Cases of extraventricular lesions, though rare, have been documented [8].

### Pathology

**Fig. 3:**
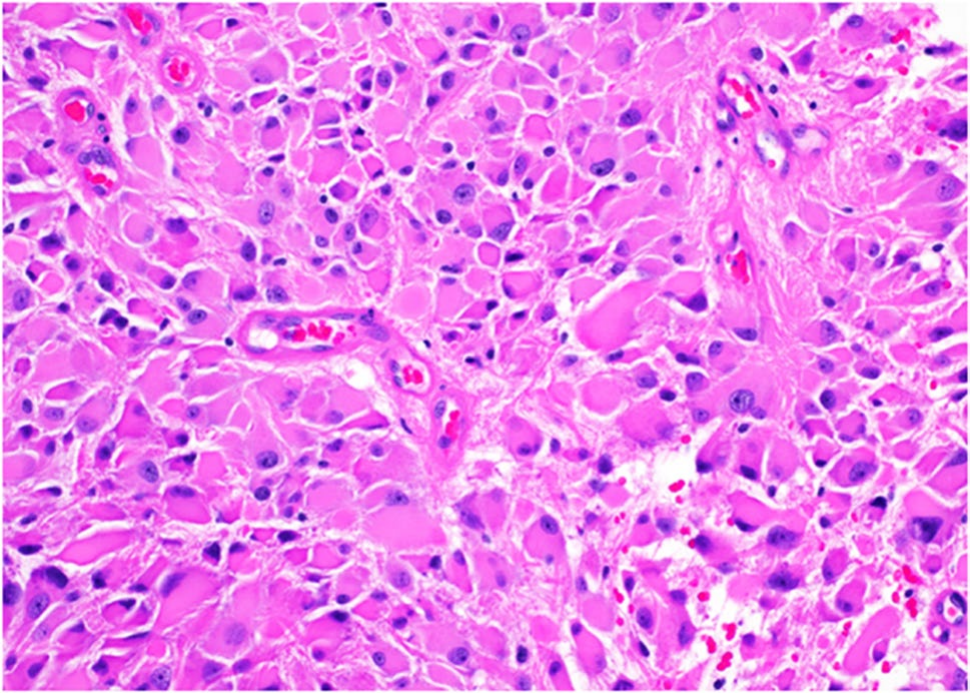
Subependymal giant cell astrocytoma in a patient with tuberous sclerosis. Image shows an astrocytic tumor composed of large cells with abundant eosinophilic cytoplasm and round nuclei with prominent nucleoli. H&E stain, 20 × magnification

Under microscopic examination, SEGAs manifest clusters or sheets of large polygonal cells. These cells are characterized by an eccentric nucleus, evenly dispersed granular chromatin, pronounced nucleoli, and a rich eosinophilic cytoplasm. Other notable features include moderate anisonucleosis and a frequent presence of binucleation and multinucleation. It is essential to note that SEGAs are distinguished by the absence of mitoses, necrosis, and vascular endothelial proliferation. These negative features underscore their benign classification (Fig. 3) [9].

### Clinical presentation

SEGAs are often identified as asymptomatic tumors [6]. When symptoms do present, they may signal increased intracranial pressure due to obstructive hydrocephalus, often stemming from a blockage at the foramen of Monro. Such symptoms can encompass headache, photophobia, diplopia, ataxia, and seizures. Additionally, those with TSC may experience behavioral disturbances and either a regression or loss of cognitive abilities [10]. Consequently, any sudden onset of symptoms in an individual known to have TSC — including headaches, visual complaints, papilledema, nausea, vomiting, or a significant increase in seizure activity — should prompt a brain imaging evaluation [8].

Issues, such as papilledema, nausea, vomiting, or a significant increase in seizure activity, should prompt a brain imaging evaluation [8].

### Key diagnostic features

For individuals with TSC, the diagnostic criteria for SEGA include the presence of a lesion near the foramen of Monro that exceeds 1 cm in any dimension at the caudothalamic groove. Additionally, any subependymal lesion, regardless of size, that displays progressive growth on consecutive imaging, regardless of its size. While SEGAs are thought to arise from subependymal nodules, this belief is debated [8]. Even SEGAs and SENs have similar histopathological features. Their differentiation is based on size and location [11]. Unlike SEGAs, SENs develop along the ependymal lining of the lateral ventricles and tend to be smaller, with reported size cutoffs ranging between 5 and 10 mm for the two types. Additionally, SENs typically exhibit little to no enhancement, while SEGAs often show significant contrast enhancement [8]. Notably, after treatment with mTOR inhibitors like Everolimus, currently considered the preferred therapeutic approach, SEGAs often exhibit a gradual decrease in size [12].

### Computerized tomography

On CT scans, SEGAs present as well-defined lesions, with an isodensity relative to the adjacent cortex. After contrast administration, these tumors often exhibit enhancement. Notably, peripheral calcification is A common feature observed in SEGA cases [13, 14].

### Magnetic resonance imaging

**Fig. 4:**
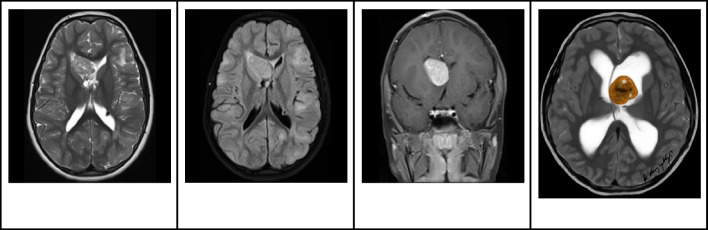
Subependymal giant cell astrocytoma in an 8-year-old boy with tuberous sclerosis complex. **A** Axial T2-weighted image shows an oval-shaped heterogeneous hyperintense nodule in the frontal horn and near the foramen of Monro. In addition, there are signs of several bilateral subcortical hyperintense hamartomas and a left periventricular subependymal nodule. **B** Axial FLAIR image shows that the nodule has smooth margins, mild mass effect, and slight hyperintense signal. In addition, the multiple bilateral subcortical hamartomas are more conspicuous than on the T2-weighted images. **C** Axial contrast-enhanced T1-weighted image shows that the mass has well-defined smooth contours and marked contrast enhancement. **D** Illustration representing a typical case of subependymal giant cell astrocytoma in the left caudothalamic groove, projecting into the left frontal horn

MRI is the recommended modality for SEGA screening due to its heightened sensitivity in detecting these lesions [8]. On MRI, SEGAs often present with an iso- to hypointense signal on T1WI and a hyperintense signal on T2WI. They also frequently exhibit pronounced and uniform contrast enhancement. Key diagnostic indicators of SEGA include a singular lesion proximate to the foramen of Monro, either a lack or partial presence of calcification, and dimensions greater than 1 cm (Fig. 4) [14, 15]. Notably, the most definitive marker remains the progressive enlargement of the lesion over time [16]. Crucially, while subependymal nodules can appear hyperintense on precontrast T1WI, they need differentiation from enhancing lesions observed on postcontrast imaging [14]. Follow-up assessments commonly utilize MRI, both with and without contrast, and are generally conducted every 1 to 3 years until the individual turns 25. Should routine imaging reveal growth in a subependymal nodule, it is recommended to contemplate more frequent follow-up imaging [8, 17].

## Central neurocytoma

### Background

CNs are infrequent, slow-growing tumors that predominantly affect adolescents and middle-aged adults. They make up roughly 0.25–0.5% of all intracranial tumors [18]. Around half of these lesions are found in the lateral ventricles near the foramen of Monro. Approximately 15% are found in both the lateral and third ventricles, and about 13% appear in both lateral ventricles along the midline. A mere 3% are found exclusively in the third ventricle, while occurrences in the fourth ventricle are scarce [19]. The term “central neurocytoma” is specifically used to describe neurocytomas within the ventricular system. In contrast, “extraventricular neurocytoma” denotes those located in the brain parenchyma, cerebellum, and spinal cord [20].

### Pathology

**Fig. 5:**
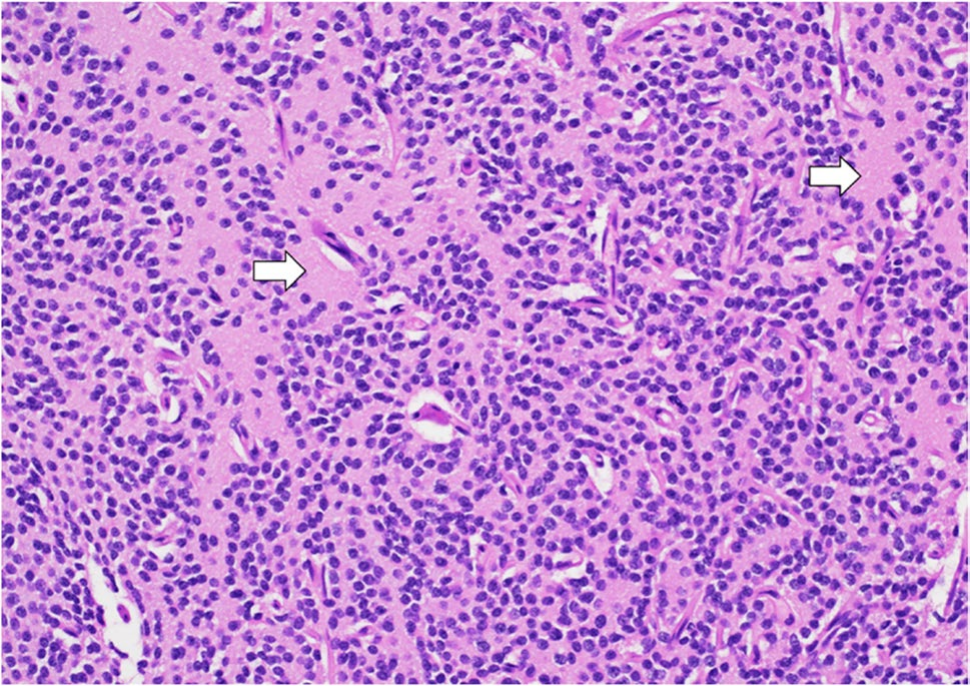
Central neurocytoma: image shows a tumor composed of monotonous cells with round nuclei and salt and pepper chromatin with islands of neuropil (arrows). H&E stain, 20 × magnification

CNs are classified as WHO grade 2 neoplasms. They typically originate from the septum pellucidum, particularly in the lower region near the foramen of Monro. Histologically, these tumors are made up of a homogeneous population of round to oval cells that have a uniform appearance. They present ultrastructural and immunophenotypic characteristics consistent with neuronal differentiation (Fig. 5). The encompassing stroma contains neuropil, which may be organized into cell-free zones that resemble rosettes [21]. In line with their neuronal lineage, CNs often exhibit strong positivity for synaptophysin and neuronal-specific enolase during immunostaining [22].

### Clinical presentation

Individuals with CNs frequently present with symptoms such as headaches, visual disturbances, nausea, and vomiting [23]. In addition, these tumors can manifest clinical indicators of increased intracranial pressure, largely due to the accompanying obstructive hydrocephalus [24, 25].

### Key diagnostic features

CNs typically present as well-circumscribed, non-invasive, lobulated masses featuring solid or cystic regions giving them a “bubbly” appearance. They are primarily situated within the lateral ventricles and are attached to the septum pellucidum [24].

### Computed tomography

On CT scans, CNs typically exhibit a hyperdense appearance. Approximately 50% of these tumors may exhibit calcification and hemorrhage components. In terms of contrast enhancement, most CNs display mild to moderate [24, 26].

### Magnetic resonance imaging

**Fig. 6:**
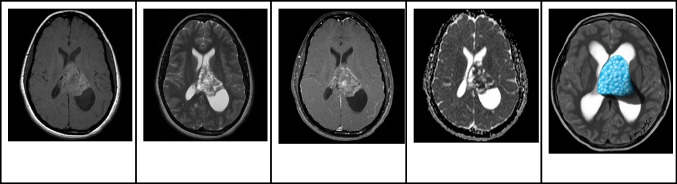
Central neurocytoma in a 17-year-old boy. **A** Axial T1 hyperintense mass in the left lateral ventricle adjacent to the septum pellucidum. **B** Axial T2-weighted image shows that the lesion is heterogeneously hyperintense with no signs of parenchymal invasion. In addition, the mass is associated with mild hydrocephalus, larger on the left side. **C** Axial contrast-enhanced T1-weighted image shows that the mass has well defined contours and heterogeneous enhancement. **D** Axial apparent diffusion coefficient image shows areas of restricted diffusion. **E** Illustration representing the case of central neurocytoma with a typical bubbly appearance

On MRI, CNs typically appear iso- to hypo-intense on T1-weighted images and iso- to hyper-intense on T2-weighted images, relative to normal brain parenchyma. Additionally, they tend to exhibit mild to moderate heterogeneous contrast enhancement [21]. On DWI, diffusion restriction within the solid component is commonly observed (Fig. 6). While the presence of cysts and the quantity of capillary-sized vessels might account for the differences in diffusion reduction, it's important to note that diffusion restriction does not correspond to proliferative potential [27]. On MRS, an increase in choline [28] or glycine may be detected [26].

## Ependymoma

### Background

Ependymomas constitute the third most prevalent brain tumor in pediatric patients, accounting for approximately 10–12% of intracranial neoplasms within this age group [29]. These tumors display a bimodal age distribution, frequently appearing in children between 1 and 5 years. They are predominantly found more often in the posterior fossa over the supratentorial regions. In adults, ependymomas are less common but, when present, often emerge during the fourth decade of life. In such cases, they typically manifest in the supratentorial compartment or the spinal canal [30, 31]. About one-third of all ependymomas arise in the supratentorial compartment, with 70% of these within the brain parenchyma. The remaining 30% are located in the ventricles, predominantly the lateral ventricles [32, 33].

### Pathology

**Fig. 7:**
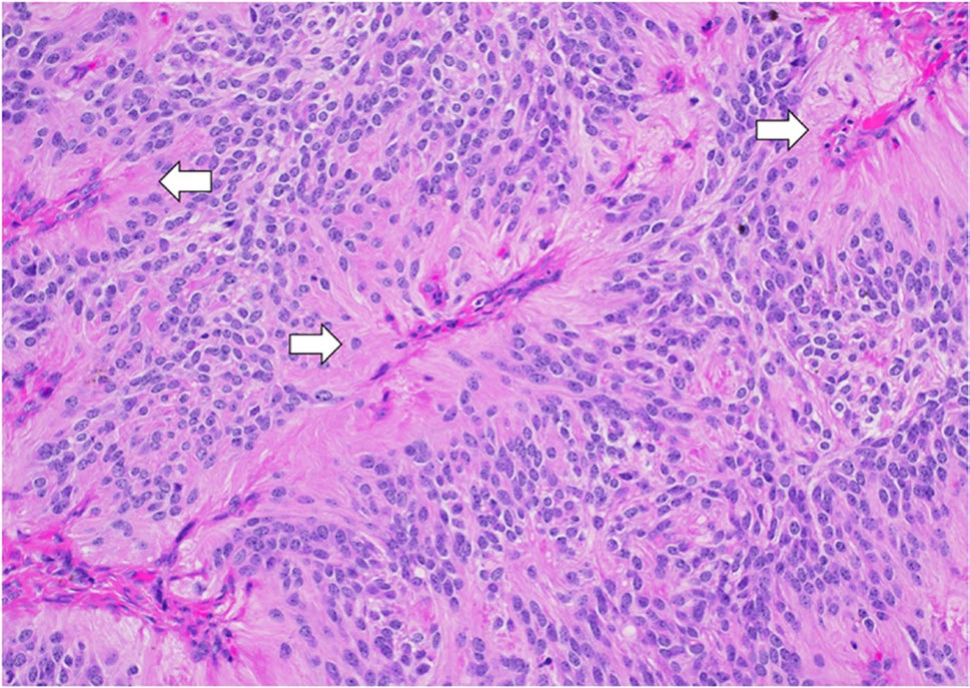
Ependymoma: image shows a glial tumor composed of tumor cells with round to ovoid nuclei with numerous perivascular pseudorosettes (arrows). H&E stain, 20 × magnification

On macroscopic examination, ependymomas appear as soft, gray-tan masses. Their hallmark histological feature is the presence of perivascular pseudorosettes, although ependymal rosettes are occasionally observed (Fig. 7) [26]. These tumors can be graded as WHO grade 2 or 3. Features indicative of high grade include increased cellularity, elevated mitotic activity, necrosis, vascular proliferation, and nuclear atypia. Although ependymomas exhibit histological similarities across the neuroaxis, supratentorial ependymomas likely comprise molecularly distinct entities. Current studies have recognized two clinically and molecularly different groups of supratentorial ependymomas: ZFTA fusion-positive and YAP1 fusion-positive. The former can occur in both children and adults and forms the majority of supratentorial ependymomas. In contrast, YAP1 fusion-positive ependymomas are mainly seen in younger children [34].

### Clinical presentation

Symptoms of supratentorial ependymomas often point to elevated intracranial pressure, manifesting as headaches, vomiting, and ataxia, frequently due to CSF flow obstruction. Additionally, these tumors can cause focal neurological deficits and seizures [26]. Notably, supratentorial intraventricular tumors often result in obstructive hydrocephalus [32].

### Key diagnostic features

The heterogeneity in imaging is the distinctive feature of ependymomas, reflecting the tumor's composition of microcysts, calcifications, and hemorrhages [33]. Intraventricular supratentorial ependymomas usually appear as smaller lesions upon diagnosis than their parenchymal equivalents. They tend to grow along the lateral ventricles’ surfaces or the septum pellucidum and frequently exhibit peripheral intratumoral cysts [32].

### Computerized tomography

On CT scans, intraventricular ependymomas present as iso- to hyper-dense masses. Between 40 and 80% of cases reveal calcification foci. Occasionally, intratumoral hemorrhage can produce a blood-fluid level. Post-contrast, these tumors typically demonstrate heterogeneous enhancement [35].

### Magnetic resonance imaging

**Fig. 8:**
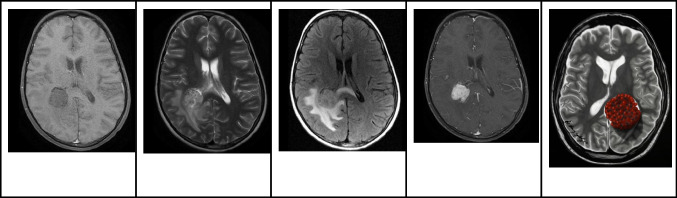
Intraventricular ependymoma in a 12-year-old girl. **A** Axial T1-weighted image shows a slightly hypointense rounded well-defined mass in the right lateral ventricle. **B** Axial T2-weighted image shows that the lesion is heterogeneously hyperintense, invading the adjacent brain and associated with adjacent edema. **C** Axial FLAIR image shows that the edema surrounding the mass is also extending through the corpus callosum.** D** Axial contrast-enhanced T1-weighted image shows that the lesion has diffuse and marked enhancement. **E** Illustration representing a case of left lateral ventricular ependymoma

On MRI, the soft-tissue component of ependymomas is typically well-circumscribed, demonstrating iso- to hypointensity on T1WI, and iso- to hyper-intensity on T2WI. The tumors also exhibit heterogeneous enhancement [26]. On DWI, ependymomas show intermediate ADC values (1.00 and 1.30 X 10^−3^ mm^2^/s) compared to other posterior fossa tumors [36] (Fig. 8). MRS often shows elevated choline and reduced NAA peaks, mirroring patterns in other brain tumors. Perfusion studies might also indicate an increased CBV in ependymomas [33].

## Cavernous malformation (cavernoma)

### Background

Cavernous malformations, also termed cavernous venous malformations or cavernomas, are CNS low-flow vascular malformations made up of clusters of dilated capillaries with no intervening brain tissue. The prevalence of cavernous malformations in infants is reported to be approximately 0.2%, with an overall prevalence of 0.6% in children. Roughly 10% of cases are familial, and around 17% demonstrate multiple lesions [37]. Cavernous malformations primarily occur in the cerebrum and basal ganglia, whereas intraventricular cavernous malformations are rare [38].

### Pathology

Microscopically, cavernous malformations appear as dilated sinusoidal channels or cavities lined by a thin endothelium, with minimal to no intervening brain tissue. These dilated vascular channels lack a muscular layer and are often surrounded by considerable hemosiderin deposition, which can be visualized on imaging. Furthermore, there is often a proliferation of the endothelium lining, along with dysfunction of the adjoining tight junctions. This can lead to leakiness, contributing to certain clinical manifestations [39].

### Clinical Presentation

The manifestation of cavernous malformations largely depends on their size and location. Intriguingly, most cavernous malformations are asymptomatic, coming to light incidentally. Yet, those located intraventricularly, especially at the foramen of Monro, are more prone to causing hydrocephalus. Common symptoms comprise headaches, nausea, and vomiting. Cranial nerve deficits and hemiparesis are less frequently observed [38, 40].

### Key diagnostic features

Radiologically, cavernous malformations often display a unique “popcorn” appearance, attributed to a central core of hemorrhage and thrombosis in various stages, encircled by a hemosiderin ring. Macroscopically, these lesions, formed from dilated capillary clusters, resemble mulberry-like structures [39].

### Computerized tomography

On CT, intraventricular cavernous malformations appear as hyperdense, non-enhancing lesions and may exhibit signs of bleeding or calcification [38].

### Magnetic resonance imaging

**Fig. 9:**
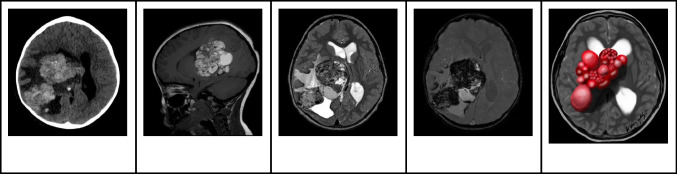
Intraventricular giant cavernous malformation in an 8-year-old boy. **A** Axial nonenhanced computed tomography image shows a large complex hyperdense hemorrhagic mass with sparse calcification foci moderate mass effect and edema. **B** Sagittal T1-weighted image shows a heterogeneous mainly lobulated multiloculated hyperintense mass in the right lateral ventricle. **C** Axial T2-weighted image shows that the lesions have a complex signal. The contours of the mass and of the thin walls of the loculations are hypointense in keeping with hemosiderin deposition. Inside the loculations, the signal is mainly hyperintense in keeping with more recent blood. In addition, there is moderate hydrocephalus and vasogenic edema surrounding the mass. **D** Axial gradient-echo image shows widespread low signal throughout the mass and in the adjacent subcortical white matter in keeping with a diffuse hemorrhage mass. **E** Illustration representing a case of right lateral cavernous malformation with multiple loculations that can undergo hemorrhage

Intraventricular cavernous malformations on MRI showcase the hallmark “popcorn” appearance, depicting multi-loculated hemorrhage and thrombosis regions encased by a hemosiderin rim. Both T1WI and T2WI project a core with a mixed signal intensity resulting from blood products of various ages. On T2WI, the surrounding rim is hypointense due to peripheral hemosiderin, forming a distinctive “black halo” [38, 41]. T1WI might show a slightly hyperintense region in the surrounding brain tissue. Hemosiderin-sensitive sequences, like gradient-recalled echo or SWI, can enhance the diagnosis by showing hypointensity at the malformation site along with blooming artifacts (Fig. 9) [39].

## High-grade gliomas

### Background

As per the WHO CNS5 classification, the term “glioblastoma” no longer falls under the category of pediatric-type diffuse HGGs [5]. Instead, glioblastomas are now classified within the group of adult-type diffuse gliomas [5]. Four pediatric types of diffuse HGGs are recognized as the following: (1) diffuse midline glioma, H3 K27-altered; (2) diffuse hemispheric glioma, H3 G34-mutant; (3) diffuse pediatric-type HGG, H3-wildtype, and IDH-wildtype; and (4) infant-type hemispheric glioma (5). Except for the infant-type hemispheric gliomas, all these tumors are grade 4. Notably, these tumors are primarily found within or may extend into the ventricular system in children. Furthermore, certain types of adult diffuse gliomas can appear in children, notably in older children and adolescents [42].

### Pathology

**Fig. 10:**
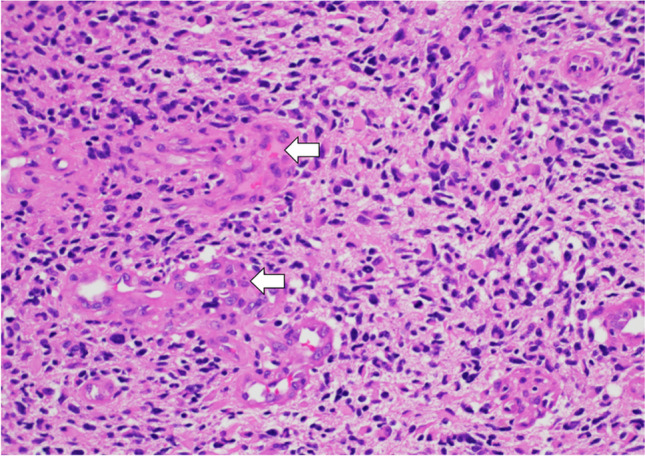
Glioblastoma histology in a tumor with extension into the right lateral ventricle: image shows a high-grade astrocytic tumor with foci of microvascular proliferation (arrows). H&E stain, 20 × magnification

HGGs, when examined microscopically, appear as highly cellular tumors with atypical nuclei and increased mitotic activity. These tumors can display necrosis, possibly surrounded by nuclear palisading. Neighboring areas might exhibit endothelial cell proliferation (Fig. 10). Immunohistochemistry shows a positive reaction to glial fibrillary acidic protein in these tumor cells [43].

### Clinical presentation

Symptoms of intraventricular HGGs manifest when their size leads to obstructive hydrocephalus or impinges on adjacent structures. Owing to their aggressive growth nature, these tumors become symptomatic relatively quickly. Common symptoms encompass headaches, visual disturbances, and indicators of elevated intracranial pressure. Less commonly, symptoms might include ataxia, motor deficits, and cognitive or psychiatric changes due to forniceal involvement [44].

### Key diagnostic features

Intraventricular HGGs display attributes consistent with HGGs located elsewhere, such as irregular, varied contrast enhancement, infiltrative, uneven edges, and necrotic sections. MRI provides superior delineation of the precise tumor location, margins, and extent compared to CT. Heterogeneous or ring-like contrast enhancement, extensive adjacent edema, and central necrosis point towards intraventricular HGG [44].

### Computerized tomography

Due to their aggressive nature, HGGs may be first identified on a CT scan in an emergency department setting. These tumors appear as irregularly shaped, iso- to hyper-dense mass lesions compared to the gray matter, with surrounding hypodensity due to infiltrating tumor and vasogenic edema. Contrast enhancement images reveal irregular, heterogeneous enhancement, along with features of central necrosis and intratumoral hemorrhage [45].

### Magnetic resonance imaging

**Fig. 11:**
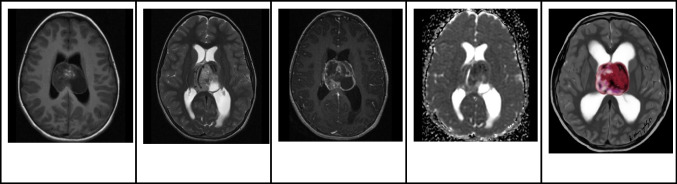
Intraventricular high-grade glioma in a 7-year-old-boy. **A** Axial T1-weighted image shows a large well defined partially hemorrhagic hypointense mass centered in the septum pellucidum involving both lateral ventricles.** B** Axial T2-weighted image shows that the lesions displaces the internal cerebral veins and has mainly hyperintense signal with areas of cystic/necrotic degeneration. **C** Axial contrast-enhanced T1-weighted image shows that the lesion has a large necrotic component with nodular peripheral enhancement. **D** Axial apparent diffusion coefficient map image shows that the majority of the lesion shows restricted diffusion. **E** Illustration representing the intraventricular high-grade glioma

MRI images of HGGs typically reveal heterogeneity, pronounced contrast enhancement, and an irregular shape. T2WI often showcases a surrounding hyperintense signal, attributable to vasogenic edema. In the presence of hemorrhage, the appearance of the tumor varies based on the timeline of bleeding. Acute and early subacute hemorrhages show iso- to hyperintensity on T1WI and a hypointense signal on T2WI (Fig. 11). The solid portions of HGGs may exhibit varying degrees of restricted diffusion. On MRS, HGGs commonly reveal elevated choline and decreased N-acetylaspartate (NAA) levels, which tend to correlate directly with increasing astrocytoma grade [45, 46].

## Pilocytic astrocytoma

### Background

PA is a slow-growing astrocytic tumor, graded as WHO CNS5 classification grade 1. Predominantly affecting children and young adults, it stands as the most common glial tumor in the pediatric demographic. In children, the cerebellum houses two-thirds of PA lesions, while in adults, half are supratentorial. In children, the most common sites of origin are the cerebellum and the region around the third ventricle, including the suprasellar region. Nevertheless, PAs can occur throughout the neuraxis [47].

### Clinical presentation

There are no clinical features unique to PAs. The signs and symptoms, typically persisting for several months, directly correlate with the tumor size, location, and the presence of associated hydrocephalus [48].

### Pathology

PAs belong to the family of circumscribed astrocytic gliomas. They are characterized by the presence of elongated, thin, highly spindle- to stellate-shaped tumor cells. These tumors exhibit a notable biphasic pattern, consisting of loose glial and compact piloid tissue. The piloid tissue component comprises dense sheets of elongated bipolar cells, displaying a distinctive feature of fine fibrillary (hair-like) processes and a typical abundance of Rosenthal fibers. In contrast, the loose glial tissue is composed of multipolar cells, microcysts, and eosinophilic granular bodies [49].

### Key diagnostic features

The features of intraventricular PAs are similar to those of PAs found in other regions of the brain. These tumors appear as lesions with well-defined margins and a mixed cystic and solid composition. The classic description is a cystic lesion with a mural enhancing nodule [49].

### Computerized tomography

Most cerebellar and cerebral PAs exhibit defined characteristics, presenting as round or oval, usually under 4 cm. These tumors frequently have cystic qualities, smooth boundaries, and sporadic calcifications. In one series, 82% of the tumors were found in close proximity to the ventricular system, and almost all (94%) demonstrated significant enhancement, typically intense, on post-contrast images obtained after the intravenous administration of contrast material [50].

### Magnetic resonance imaging

**Fig. 12:**
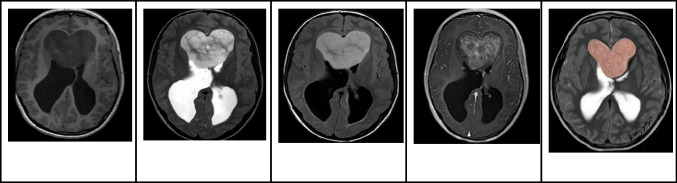
Intraventricular pilocytic astrocytoma in an 8-year-old boy. **A** Axial T1-weighted image shows a large well defined hypointense mass centered in the anterior septum pellucidum involving both lateral ventricles.** B** Axial T2-weighted image shows that the lesion fills the bilateral frontal horns has mainly hyperintense signal with hypointense strands. **C** Axial FLAIR image shows that the lesion has a fairly homogeneous hyperintense signal with no signs of invasion or edema of the adjacent brain parenchyma. **D** Axial contrast-enhanced T1-weighted image shows that the majority of the lesion shows whorls of heterogeneous enhancement. **E** Illustration representing the intraventricular pilocytic astrocytoma

On MRI, PAs present as well-circumscribed tumors, and their imaging features are influenced by their composition. They often present with a combination of solid and cystic components. The cystic portions of PAs display pronounced T2 hyperintensity and T1 hypointensity, similar to that of CSF, with or without wall enhancement. The solid components demonstrate moderate T2 hyperintensity and T1 hypointensity, along with vivid enhancement (Fig. 12). In DWI, no restricted diffusion is noted [49].

## Ganglioglioma

### Background

Gangliogliomas are uncommon CNS tumors, comprising both glial and differentiated neuronal elements. They account for approximately 0.4–7.6% of all primary brain tumors, predominantly manifesting in children and young adults under 30 years of age. The most common locations for these tumors are the temporal lobe and cerebellum, although they can develop anywhere within the CNS [51]. A comprehensive review highlighted that only about 3.74% of all gangliogliomas emerge within the ventricles [52].

### Pathology

Microscopically, gangliogliomas are defined by the coexistence of dysmorphic neuronal cells and a neoplastic glial population. Eosinophilic granular bodies and perivascular inflammation are often present. Typically, gangliogliomas align with a WHO grade 1 classification in the CNS [53, 54].

### Clinical presentation

Headaches are the predominant symptom for intraventricular gangliogliomas. Other manifestations can encompass dizziness, vomiting, visual disturbances, seizures, and papilledema [51]. Notably, gangliogliomas in the temporal lobe, their most common site, frequently correlate with seizures and epilepsy [55].

### Key diagnostic features

Despite the rarity of intraventricular gangliogliomas, they should be included in the differential diagnosis of intraventricular tumors. Their imaging characteristics can vary, and they often lack the hallmark traits of extra-ventricular gangliogliomas, such as a prominently enhancing mural nodule [56, 57]. Therefore, a definitive diagnosis of ganglioglioma cannot be established solely relying on imaging [57, 58].

### Computerized tomography

On CT scans, which are typically conducted during the initial examination, gangliogliomas may present as hypodense, mixed hypodense/isodense, solid, or partially cystic masses [59].

### Magnetic resonance imaging

**Fig. 13:**
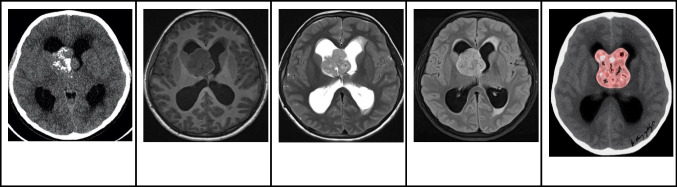
Intraventricular ganglioglioma in a 6-year-old boy. **A** Axial non-enhanced contrast computed tomography shows a partially calcified mass in the right frontal horn. **B** Axial T1-weighted image shows that the lesion is diffusely hypointense, and deviates the anterior septum pellucidum medially. **C** Axial T2-weighted image shows that the lesion is diffusely hyperintense with an eccentric focus of cystic degeneration. **D** Axial FLAIR image shows that the lesion has a fairly homogeneous hyperintense signal with no signs of invasion or edema of the adjacent brain parenchyma. In addition, there is moderate hydrocephalus and interstitial edema. **E** Illustration representing an intraventricular ganglioglioma

On MRI, intraventricular gangliogliomas often lack the typical features seen in extra-ventricular gangliogliomas. Extra-ventricular gangliogliomas typically present as a cyst with an enhancing mural nodule, and they exhibit characteristics of low-grade lesions, such as well-defined margins without surrounding infiltration. They display iso- or hypointensity on T1WI and hyperintensity on T2WI. Although contrast enhancement is observed, it is variable and may be nodular, rim-like, or entirely solid. In approximately 50% of cases, cyst formation accompanied by mural nodules and calcifications can be noted [53, 59]. Diagnosing intraventricular gangliogliomas solely based on imaging is not typically feasible due to their considerable variability [57, 58]. Approximately 40–70% of gangliogliomas exhibit isointensity while 20–40% appear hypointense on T1-weighted imaging. On T2-weighted imaging, around 20–30% of gangliogliomas present as isointense, whereas 70–90% display hyperintensity (Fig. 13) [58, 60].

## Myxoid glioneuronal tumor, PDGFRA P.K385-mutant

### Background

MGNTs are recognized as low-grade tumors and belong to the broader family of glioneuronal and neuronal tumors [5]. Predominantly localized along the septum pellucidum and septal nuclei, they are less often encountered within the corpus callosum. A defining feature of these tumors is a particular mutation in the PDGFRA oncogene [61].

### Pathology

Under microscopic scrutiny, MGNTs display low-grade proliferation, with histological patterns akin to dysembryoplastic neuroepithelial tumors (DNTs) or rosette-forming glioneuronal tumors (RGNTs). The tumors consist of oligodendrocyte-like cells set within a pronounced myxoid stroma. Distinguishingly, they do not present the mucin-patterned nodules characteristic of cortically based DNETs. MGNTs are also devoid of the BRAF and FGFR1 mutations or rearrangements observed in other low-grade neuroepithelial tumors [62]. These tumors can exhibit a methylation profile similar to that of DNTs. Their histological presentation includes a delicate capillary network reminiscent of DNTs or oligodendrogliomas and may occasionally display neurocytic rosettes. These tumors do not present features such as mitosis, necrosis, or glomeruloid microvascular proliferation [62]. Their unique positioning in the septum pellucidum and lateral ventricle distinctly separates them from DNTs and RGNTs, which are characteristically located in the cortical and fourth ventricular regions, respectively [61].

### Clinical presentation

The predominant symptom among affected individuals is intermittent headaches. The clinical spectrum also includes seizures, vomiting, behavioral changes, visual anomalies, altered mental states, hydrocephalus, and personality shifts. Some patients, however, remain asymptomatic, with the tumor being identified incidentally during neuroimaging evaluations [63].

## Key diagnostic features

The primary imaging characteristic of this tumor is its location. MGNTs typically involve the septum pellucidum and margins of the lateral ventricles, appearing as a non-enhancing nodular mass [63].

### Computerized tomography

On CT scans, MGNT presents as a hypodense, well-delineated lesion with a cyst-like or pseudocyst appearance [63].

### Magnetic resonance imaging

**Fig. 14:**
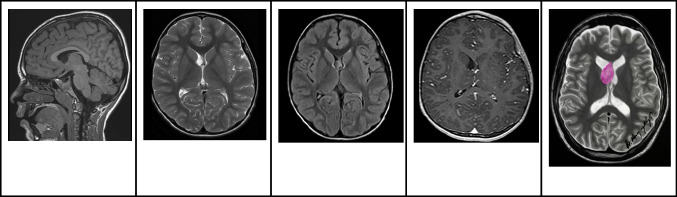
Myxoid glioneuronal tumor, PDGFRA p.K385-mutant in a 10-year-old girl. **A** Sagittal T1-weighted image shows a discrete rounded hypointense mass. **B** Axial T2-weighted image shows that the lesion is diffusely hyperintense, seen along the septum pellucidum and sitting near the right caudothalamic groove. **C** Axial FLAIR image shows that the lesion has a hyperintense rim with no signs of edema or invasion. **D** Axial contrast-enhanced T1-weighted image shows that the lesion has no signs of enhancement. **E** Illustration representing a myxoid glioneuronal tumor, PDGFRA p.K385-mutant

MRI reveals a well-defined, lobulated mass that is hypointense on T1WI, hyperintense on T2WI, and shows no enhancement with contrast. FLAIR imaging is crucial in the diagnostic process, showcasing a partially suppressed central signal and an encircling peripheral hyperintense rim, a pattern synonymous with DNETs. Additionally, DWI indicates facilitated diffusion. Furthermore, DWI display facilitated diffusion (Fig. 14) [64]. In sporadic instances, complications like obstructive hydrocephalus and ventricular dissemination may be evident [62, 63].

## Embryonal tumors with multilayered rosettes

### Background

Embryonal tumors with multilayered rosettes (ETMR) are rare, highly malignant embryonal tumors originating from the primitive neuroectodermal epithelium that lines the neural tube [65]. In the past, these tumors were known by various names, including embryonal tumors with abundant neuropil and true rosettes (ETANTR), ependymoblastoma, and medulloepithelioma. However, recent genetic and molecular data suggest that these different classifications represent a single clinicopathological entity [66]. The peak incidence of ETMR is observed between the ages of 6 months and 5 years. These tumors can occur anywhere within the CNS, but they are most commonly found in the periventricular region, specifically in the temporal and parietal lobes and the brainstem. Cases have also been reported within the ventricles. Owing to their high degree of malignancy, these tumors generally have a poor prognosis, with a 5-year survival rate ranging between 0 and 30% [66, 67].

### Pathology

ETMR, C19MC-altered is a recently defined entity and is classified as a grade 4 tumor [68]. The second most common genetic variant of these tumors exhibits biallelic mutations in DICER1. The tumor consists of pluripotent stem cells that can differentiate into various types of cells such as mesenchymal cells, neurons, astrocytes, ependymal cells, or oligodendrocytes, as detected by immunohistochemistry. These cells may be arranged in tubules, trabeculae, or papillae, resembling the structure of the primitive neural tube. Additionally, necrosis and hemorrhage have been observed in histological studies of these tumors [69, 70].

### Clinical presentation

ETMRs in the intraventricular region commonly present with obstructive hydrocephalus. Given that they primarily occur in infants and young children, frequent symptoms include persistent vomiting and an enlarged head circumference [67].

## Key diagnostic features

Many of the imaging characteristics of ETMRs are similar to those of other common intraventricular tumors. Therefore, definitive confirmation of diagnosis can typically only be achieved through a molecular examination for C19MC alterations [71].

### Computerized tomography

On CT, ETMRs present as hypo- or iso-dense masses with well-defined borders [67].

### Magnetic resonance imaging

**Fig. 15:**
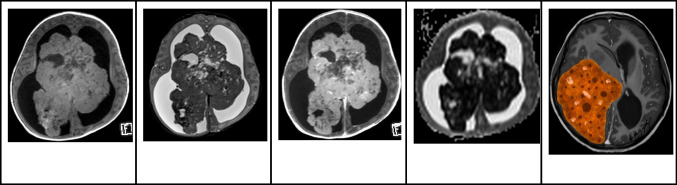
Intraventricular ETMR in a 1-year-old boy. **A** Axial T1-weighted image shows a large macrolobulated predominantly hyperintense mass centered in the midline. **B** Axial T2-weighted image shows that the mass is diffusely hypointense with central and peripheral cystic/necrotic degeneration. In addition, the lesion projects bilaterally to both lateral ventricles with marked hydrocephalus. **C** Axial contrast-enhanced T1-weighted image shows that the lesion has moderate and heterogeneous enhancement. **D** Axial apparent diffusion coefficient map image shows that the lesion has marked restricted diffusion. **E** Illustration representing an ETMR

On MRI, these tumors typically appear as iso- or hypo-intense on T1WI, and hyperintense on T2WI. Approximately 95% of these tumors will exhibit contrast enhancement, particularly within the solid elements. The enhancement may often display heterogeneity with an uneven distribution, which can be attributed to tumor features such as necrosis and blood vessel formation (Fig. 15) [70]. Additionally, these tumors typically demonstrate restricted diffusion, an indication often associated with high cellularity [71].

## Teratoma

### Background

Teratomas are rare CNS germ cell tumors representing around 0.5% of all intracranial tumors [72]. However, they are the most prevalent CNS tumors present at birth, accounting for between 28.8 and 50% of all congenital brain tumors [73]. While teratomas are typically found in the sacrococcygeal region, they can also occur in the chest, neck, and intracranially [74]. Within the brain, they predominantly arise from midline structures, notably the pineal and suprasellar regions [75, 76]. Intraventricular teratomas are exceedingly rare, with only a few reported cases [77].

### Clinical presentation

The majority of teratomas are large upon presentation, often associated with increased intracranial pressure, macrocephaly, or hydrocephalus. Some affected children may unfortunately be stillborn. The prognosis of congenital intracranial teratomas is generally unfavorable due to their substantial size, rich blood supply, and extensive invasion [78].

### Pathology

Teratomas can be classified into three categories: mature teratomas, immature teratomas, and teratomas with somatic-type malignancy. Mature teratomas contain only fully differentiated, adult-type tissue elements that exhibit little, if any, mitotic activity. They often consist of solid components, cysts of diverse sizes, potential mucinous material, calcified areas, and chondroid nodules, typically excluding hemorrhage and necrosis [79]. Immature teratomas, on the other hand, are characterized by the presence of even small tissue components that have incompletely differentiated, resembling fetal structures. These tumors may contain cysts, calcifications, and chondroid material, but they generally have soft, fleshy components reflecting the high cellularity of immature elements [80]. Teratomas with somatic-type malignancy can mirror mature or immature teratomas but are more likely to exhibit regional necrosis and possible overgrowth by sarcomatous components [81].

### Key diagnostic features

The imaging appearance of teratomas is highly variable, typically presenting as a well-circumscribed, lobulated, heterogeneous mass. This heterogeneity is attributed to the presence of elements such as calcification, fat, cysts, hemorrhage, and solid components. Heterogeneous contrast enhancement may also be observed [82].

### Computerized tomography

Teratomas, on CT, typically present as heterogeneous masses with a mix of different densities. If present, fatty components are markedly hypodense with negative Hounsfield units and can provide helpful clues for diagnosis. Cystic components may exhibit hypo-, iso-, or hyper-density depending on the presence of hemorrhagic and proteinaceous material. Components with increased density may represent soft tissue interspersed with hemorrhagic components, whereas extremely dense components may indicate calcification [82].

### Magnetic resonance imaging

**Fig. 16:**
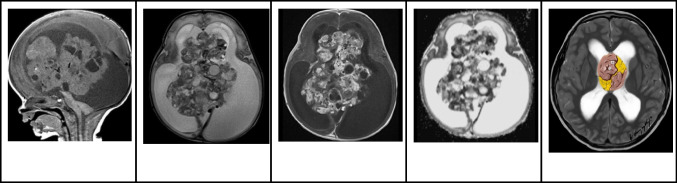
Intraventricular teratoma in a newborn boy, born with severe macrocephaly

On MRI, teratomas typically present as well-defined masses with heterogeneous signal intensity on T1- and T2-WI. Both fatty and high proteinaceous material usually appear hyperintense on T1WI. In contrast, calcification and blood products are typically hypointense on both T1WI and T2WI. Cystic, solid, and hemorrhagic components demonstrate heterogeneous iso- to hyper-intense T2 signals. Contrast enhancement, typically seen in the soft tissue components, is usually heterogeneous (Fig. 16) [82–84].

## Conclusion

Examination of tissue samples is essential for an accurate diagnosis and appropriate treatment planning for supratentorial intraventricular tumors in children. Crucial management decisions often hinge on determining which lesions are amenable to surgical intervention. Detailed preoperative neuroimaging plays a pivotal role in this surgical planning. Recognizing the typical imaging characteristics of these tumors can assist in making a preliminary diagnosis when tissue sampling is unavailable, as a select few of these lesions might be managed with watchful waiting.

The intraventricular masses originate from different locations, such as the choroid plexus, the ependymal lining of the lateral ventricles, the subependymal plate of the ventricular wall, glial-lined structures like the septum pellucidum, paraventricular structures, or adjacent vessels. While primary choroid plexus tumors (CPPs, aCPPs, and CPCs) are the most common and clinically relevant supratentorial intraventricular masses in children, it is important to consider other types of tumors in the differential diagnosis.

Astrocytomas typically emerge either midline, originating from the septum pellucidum or fornices, or intra-axially with a ventricular extension. Imaging features such as invasion, surrounding brain edema, and CSF seeding are typical imaging features of high-grade tumors, encompassing CPCs, ATRTs, and embryonal tumors.

A complete imaging scan of the entire neuraxis might be crucial to detect CSF seeding. Prominent vascularity with apparent flow voids might suggest the presence of a vascular malformation. However, it Is essential to realize that a plethora of other masses can also show heightened vascularity. The absence of enhancement is a hallmark in conditions like colloid cysts and myxoid glioneuronal tumors. Ependymomas, in certain instances, can appear solely as intraventricular masses.

Surgical intervention stands out as the pivotal treatment method for supratentorial intraventricular masses in pediatric patients. Hence, achieving a precise preoperative diagnosis becomes indispensable for both surgical preparation and staging procedures. Our comprehensive framework delivers a structured methodology for addressing these tumors, facilitating a thorough and increasingly precise preoperative assessment of supratentorial intraventricular masses in children.
